# Comparative effectiveness of telmisartan vs. other angiotensin receptor blockers in reducing hypertension-related cerebrovascular and cardiovascular events: a real-world retrospective study using the TriNetX network

**DOI:** 10.3389/fcvm.2025.1715032

**Published:** 2026-01-22

**Authors:** Tse-Yu Chen, Yi-Chun Lin, Guang-Yaw Liu, Hui-Chih Hung

**Affiliations:** 1 Doctoral Program in Translational Medicine, National Chung Hsing University, Taichung, Taiwan; 2 Department of Neurosurgery, Neurological Institute, Taichung Veterans General Hospital, Taichung, Taiwan; 3 Rong Hsing Translational Medicine Research Center, National Chung Hsing University, Taichung, Taiwan; 4 Department of Life Sciences, National Chung Hsing University, Taichung, Taiwan; 5 Institute of Medicine, College of Medicine, Chung Shan Medical University, Taichung, Taiwan; 6 Division of Allergy, Immunology and Rheumatology, Department of Medicine, Chung Shan Medical University Hospital, Taichung, Taiwan; 7 iEGG and Animal Biotechnology Center, National Chung Hsing University, Taichung, Taiwan; 8 Advanced Plant and Food Crop Biotechnology Center, National Chung Hsing University, Taichung, Taiwan

**Keywords:** Telmisartan, angiotensin receptor blockers, hypertension, stroke, mortality, real-world evidence

## Abstract

**Introduction:**

Telmisartan is a long-acting angiotensin II receptor blocker (ARB) with unique pharmacologic properties, including partial PPAR-γ activation. Its comparative effectiveness against other ARBs in real-world populations remains unclear.

**Methods:**

We conducted a retrospective cohort study using the TriNetX Global Collaborative Network, including hypertensive patients aged 55–85 years without prior stroke, heart failure, or myocardial infarction. After 1:1 propensity score matching, 41,598 patients were included in each group.

**Results:**

Telmisartan use was associated with a significantly lower risk of stroke (HR 0.805, 95% CI 0.751–0.863), heart failure (HR 0.75, 95% CI 0.672–0.836), and all-cause mortality (HR 0.59, 95% CI 0.542–0.642) compared to other ARBs. Subgroup analyses showed consistent benefits across sex, diabetes, chronic kidney disease, and hyperlipidemia.

**Conclusions:**

In this large real-world matched cohort of over 83,000 patients, telmisartan was associated with superior cardiovascular and cerebrovascular outcomes compared to other ARBs, supporting its potential as a preferred antihypertensive agent in high-risk populations.

## Practical applications

This real-world evidence study, involving over 2 million patients, suggests that telmisartan may offer additional protective benefits beyond blood pressure control—particularly in reducing the risk of stroke, heart failure, and all-cause mortality—compared to other commonly used ARBs. Given its long-acting profile and unique anti-inflammatory and metabolic properties, telmisartan may be especially beneficial for patients with hypertension who also have diabetes, chronic kidney disease, or dyslipidemia. These findings have meaningful implications for clinicians, pharmaceutical decision-makers, and public health professionals seeking effective, well-tolerated treatments for high-risk hypertensive populations. By informing drug selection based on long-term cardiovascular outcomes, this research supports the personalized optimization of antihypertensive therapy in routine care.

## Introduction

Hypertension is a well-established risk factor for cardiovascular disease and has been consistently linked to increased risks of coronary artery disease, heart failure, stroke, and all-cause mortality in large-scale epidemiological studies (1). The Multi-Ethnic Study of Atherosclerosis (MESA) further demonstrated that individuals with both hypertension and elevated lipoprotein(a) levels have a significantly greater risk of cardiovascular events compared to those without hypertension (2). A broad range of antihypertensive agents—including calcium channel blockers (3), beta-blockers (4), angiotensin-converting enzyme inhibitors (ACEIs), and angiotensin II receptor blockers (ARBs) (5)—are routinely employed in clinical practice to reduce hypertension-related cardiovascular morbidity and mortality.

Telmisartan is a highly selective angiotensin II type 1 (AT1) receptor blocker approved for the treatment of hypertension (6), either in monotherapy or in combination with other antihypertensive agents (7, 8). Its long elimination half-life ensures sustained 24-hour blood pressure control, supporting its role as an effective first-line therapy for essential hypertension (9, 10). Clinical trials have demonstrated telmisartan's sustained efficacy, and it is well tolerated across a broad range of patient populations, including the elderly and individuals with comorbid conditions such as type 2 diabetes and renal impairment (11–13). Additionally, telmisartan has been associated with improvements in insulin sensitivity (11, 14, 15), lipid metabolism (16), and may offer potential neuroprotective benefits (17–19).

While several studies have highlighted the potential superiority of telmisartan in reducing hypertension-related cardiovascular events, recent real-world evidence suggests that its cardiovascular outcomes may be comparable to those of other ARBs in hypertensive patients (20), although additional real-world data are warranted to further strengthen claims of its superior cardiovascular benefits. This study aims to evaluate the cardiovascular protective effects of telmisartan relative to other ARBs using real-world evidence.

## Methods

### Data source

This retrospective cohort study utilized the TriNetX platform, a global federated health research network that provides access to deidentified electronic health records from numerous large healthcare organizations (HCOs). The platform integrates data from both electronic health records and insurance claims into a comprehensive longitudinal dataset. Available information includes patient demographics, diagnoses [coded using the International Classification of Diseases, Tenth Revision, Clinical Modification (ICD-10-CM)], procedures [using ICD-10 Procedure Coding System (ICD-10-PCS) and Current Procedural Terminology (CPT)], medications (coded via the Veterans Affairs National Formulary), laboratory results [coded using Logical Observation Identifiers Names and Codes (LOINC)], and healthcare utilization metrics.

The data for this study were obtained on July 12, 2025, from the TriNetX Global Collaborative Network, which is comprised of 147 HCOs and includes records from over 170 million patients. Further methodological details and validation of the platform have been described in prior publications (21, 22).

### Ethics statement

All data used in this study were de-identified, thereby exempting the requirement for informed consent. The TriNetX platform complies with the Health Insurance Portability and Accountability Act (HIPAA) and the General Data Protection Regulation (GDPR). Additionally, this study was approved by the Institutional Review Board (IRB) of our institution.

### Study design

**Figure 1:**
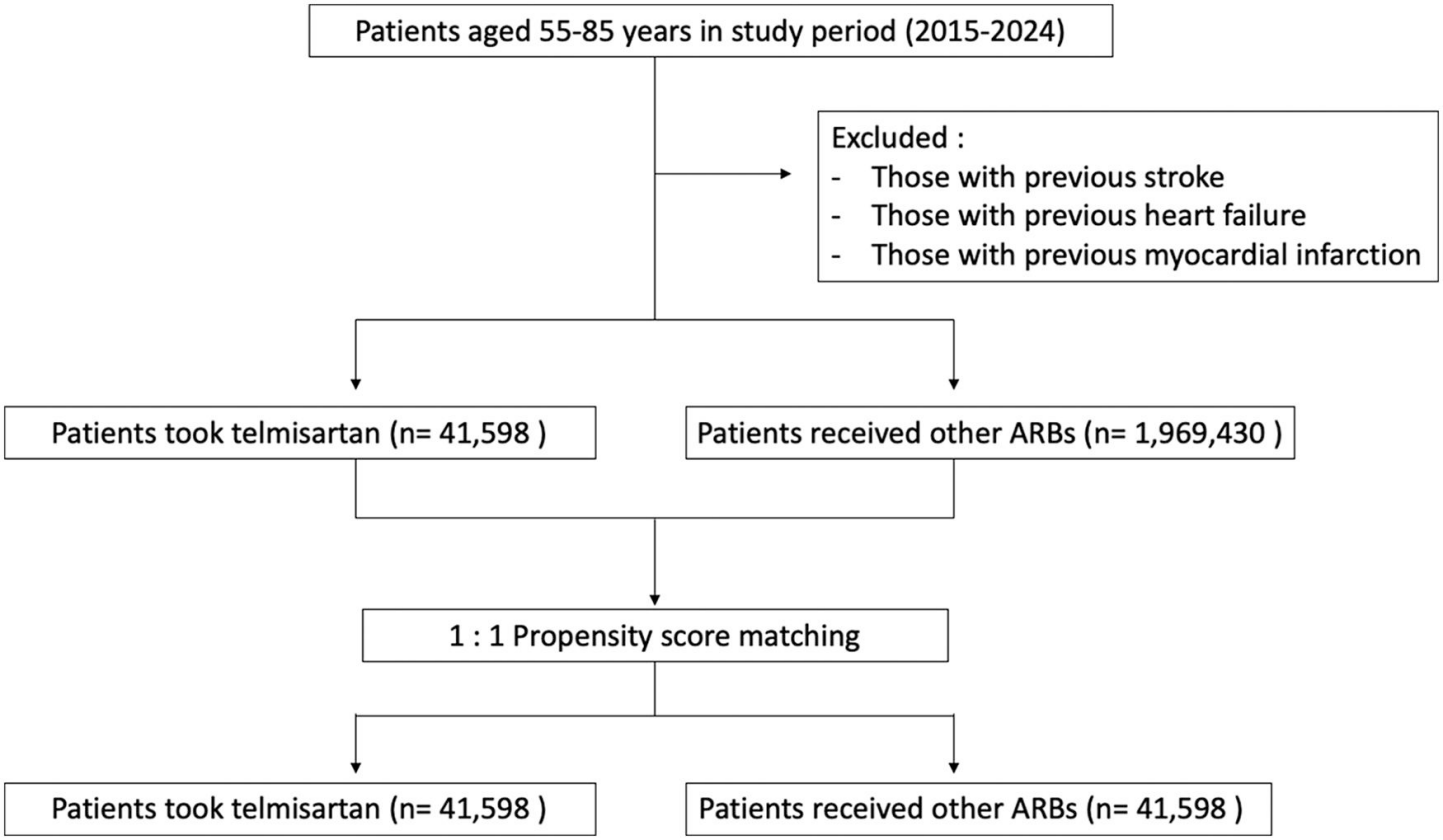
Study design and cohort construction.

The cohort construction process and patient selection criteria are illustrated in Figure 1. Patients who received telmisartan without exposure to any other ARBs (azilsartan, candesartan, olmesartan, losartan, valsartan, eprosartan, or irbesartan) and had a documented refill of telmisartan after two months of the initial prescription, were assigned to the “telmisartan” cohort. Patients who received losartan without exposure to any other ARBs (azilsartan, candesartan, olmesartan, valsartan, eprosartan, telmisartan or irbesartan), and had a documented refill of losartan after two months of the initial prescription, were assigned to the “losartan” cohort. The “Valsartan” cohort was established accordingly with patients exclusively receiving valsartan and had a documented refill of valsartan after two months of the initial prescription. Those who received ARBs other than telmisartan were classified into the “other ARBs” cohort. The date of the first prescription was defined as the index event. Patients younger than 55 or older than 85 years, as well as those with a prior history of heart failure, stroke, or myocardial infarction, were excluded from the analysis.

The primary outcomes of interest were stroke (both ischemic and intracranial hemorrhage, ICD-10-CM: G46, I60 −I63, I65 −I67, I69; ICD-9-CM: 433, 433.0 −433.9, 434, 434.0, 434.1, 434.9), coronary heart disease (ICD-10-CM: I20-I25), heart failure (ICD-10-CM: I50) and all-cause mortality. The observation period began one day after the initial administration of the drug (the index event) and extended up to five years post-administration.

### Statistical analysis and data visualization

Propensity score matching (PSM) was performed using the built-in functionality of the TriNetX platform to create 1:1 matched cohorts based on selected covariates. The matching process utilized a greedy nearest-neighbor algorithm with a caliper of 0.1 pooled standard deviations, ensuring a maximum allowable difference in propensity scores of less than 0.1. Matching variables included age at index date, sex (male), and ethnicity (Hispanic or Latino, White, Asian, Black or African American, Native Hawaiian or Other Pacific Islander, American Indian or Alaska Native). Additional clinical and treatment-related variables included essential hypertension (ICD-10-CM: I10), secondary hypertension (ICD-10-CM: I15), disorders of lipoprotein metabolism and other lipidemias (ICD-10-CM: E78), chronic kidney disease (ICD-10-CM: N18), and baseline use of the following medications: beta-blockers (VA: CV100), calcium channel blockers (VA: CV200), diuretics (VA: CV700), ACE inhibitors (VA: CV800), antiarrhythmics (VA: CV300), antianginals (VA: CV250), oral hypoglycemic agents (VA: HS502), platelet aggregation inhibitors (VA: BL117), warfarin (RxNorm: 11289), and other anticoagulants (VA: BL110).

From the TriNetX Analytics platform, survival analyses were conducted using time-to-event data. Kaplan–Meier (KM) survival curves were constructed to estimate the cumulative incidence of outcomes over time, stratified by treatment cohorts (e.g., telmisartan vs. other ARBs). For visual clarity, KM curves were plotted with time expressed in years (converted from days), and survival probabilities were displayed on the *y*-axis. The *x*-axis was limited to 5 years of follow-up, and the *y*-axis was scaled to highlight differences near the upper survival range (e.g., 0.60–1.00). Forest plots were generated to compare hazard ratios (HRs) and 95% confidence intervals (CIs) for clinical outcomes between cohorts. HRs were derived using Cox proportional hazards models available on the TriNetX platform, with other ARBs serving as the reference group. Forest plots were created without logarithmic scaling, and *x*-axis limits were adjusted to optimize visibility of the CI ranges. Point estimates were represented by symbols and horizontal lines indicating the 95% CI, with annotations displaying the exact HR and CI values. All plots were produced using Python (v3.10) with the matplotlib and pandas libraries.

## Results

### Baseline characteristics

A total of 41,598 patients were included in the telmisartan cohort, and 1,969,430 patients were included in the comparator group receiving other angiotensin II receptor blockers (ARBs), following the exclusion of individuals with prior stroke, heart failure, or myocardial infarction (Table 1). Patients in the telmisartan group were more likely to be of Asian ethnicity, whereas a greater proportion of Black or African American individuals were observed in the other ARBs group. Baseline age and sex distribution were comparable between cohorts. Regarding concomitant medications, the telmisartan cohort had a higher prevalence of platelet aggregation inhibitor use, while patients in the other ARBs group more frequently received antiarrhythmics and anticoagulants.

**Table 1:** Baseline characteristics of the study cohort before and after propensity score matching.

	Initial group						Propensity score-matched group					
	Telmisartan (*n* = 41,598)		Other ARBs (*n* = 1,969,430)		*p* value	SMD	Telmisartan (*n* = 41,598)		Other ARBs (*n* = 41,598)		*p* value	SMD
Demographics												
Age at baseline (years)	64.5 ± 8.11		64.2 ± 8.25		< 0.0001	0.04	64.5 ± 8.11		64.5 ± 8.14		0.38	0.01
Male (%)	21,297	51.20%	889,491	45.17%	< 0.0001	0.12	21,297	51.20%	21,309	51.23%	0.73	0.00
Race (*n*, %)												
White	17,063	41.02%	1,186,972	60.27%	< 0.0001	0.39	17,063	41.02%	17,098	41.10%	0.81	0.00
Asian	6,448	15.50%	128,078	6.50%	< 0.0001	0.29	6,448	15.50%	6,268	15.07%	0.08	0.01
Black or African American	3,698	8.89%	252,134	12.80%	< 0.0001	0.13	3,698	8.89%	3,687	8.86%	0.89	0.00
Hispanic or Latino	1,736	4.17%	102,852	5.22%	< 0.0001	0.05	1,736	4.17%	1,679	4.04%	0.32	0.01
American Indian or Alaska Native	96	0.23%	5,244	0.27%	0.16	0.01	96	0.23%	103	0.25%	0.62	0.00
Native Hawaiian or Other Pacific Islander	86	0.21%	7,633	0.39%	< 0.0001	0.03	86	0.21%	87	0.21%	0.94	0.00
Diagnosis (*n*, %)												
Disorders of lipoprotein metabolism	8,515	20.47%	540,197	27.43%	< 0.0001	0.16	8,515	20.47%	8,560	20.58%	0.70	0.00
Chronic kidney disease (CKD)	1,385	3.33%	86,904	4.41%	< 0.0001	0.06	1,385	3.33%	1,297	3.12%	0.08	0.01
Medication uses (*n*, %)												
Antilipemic agents	9,845	23.67%	461,435	23.43%	0.26	0.01	9,845	23.67%	9,717	23.36%	0.30	0.01
Calcium channel blockers	5,877	14.13%	281,627	14.30%	0.32	0.00	5,877	14.13%	5,786	13.91%	0.36	0.01
Beta blockers	5,633	13.54%	302,358	15.35%	< 0.0001	0.05	5,633	13.54%	5,536	13.31%	0.32	0.01
Oral hypoglycemic agents	4,764	11.45%	214,089	10.87%	< 0.001	0.02	4,764	11.45%	4,648	11.17%	0.20	0.01
Diuretics	4,743	11.40%	298,284	15.15%	< 0.0001	0.11	4,743	11.40%	4,680	11.25%	0.49	0.00
Platelet aggregation inhibitors	4,229	10.17%	167,048	8.48%	< 0.0001	0.06	4,229	10.17%	4,155	9.99%	0.39	0.01
ACE inhibitors	2,810	6.76%	193,014	9.80%	< 0.0001	0.11	2,810	6.76%	2,600	6.25%	0.00	0.02
Antiarrhythmics	2,693	6.47%	210,636	10.70%	< 0.0001	0.15	2,693	6.47%	2,555	6.14%	0.05	0.01
Anticoagulants	2,275	5.47%	148,904	7.56%	< 0.0001	0.08	2,275	5.47%	2,056	4.94%	0.00	0.02
Antianginals	1,638	3.94%	59,765	3.04%	< 0.0001	0.05	1,638	3.94%	1,560	3.75%	0.16	0.01
Warfarin	175	0.42%	15,767	0.80%	< 0.0001	0.05	175	0.42%	148	0.36%	0.13	0.01

ARB, Angiotensin receptor blockers; SMD, standardized mean difference.

After propensity score matching, 41,598 patients were retained in each cohort for the analyses of heart failure and all-cause mortality. For stroke and coronary artery disease (CAD), the final analytic populations were slightly smaller due to differences in data availability and endpoint completeness (Table 2).

**Table 2:** Major clinical outcomes comparing telmisartan and other ARBs.

Events	Total number for analysis	5-year cumulative rate (%)	Hazard ratio, 95% CI	*P*-value
Heart failure				
Other ARBs	41,598	801 (1.926%)	1.0	Ref.
Telmisartan	41,598	540 (1.298%)	0.75 (0.672,0.836)	< 0.0001
Stroke				
Other ARBs	40,026	1,868 (4.667%)	1.0	Ref.
Telmisartan	40,219	1,383 (3.439%)	0.805 (0.751,0.863)	< 0.0001
Coronary artery disease				
Other ARBs	36,331	3,924 (10.801%)	1.0	Ref.
Telmisartan	36,376	3,529 (9.701%)	0.966 (0.923,1.011)	0.1314
All-cause death				
Other ARBs	41,538	1,529 (3.681%)	1.0	Ref.
Telmisartan	41,524	810 (1.951%)	0.59 (0.542,0.642)	< 0.0001

### Heart failure

**Figure 2:**
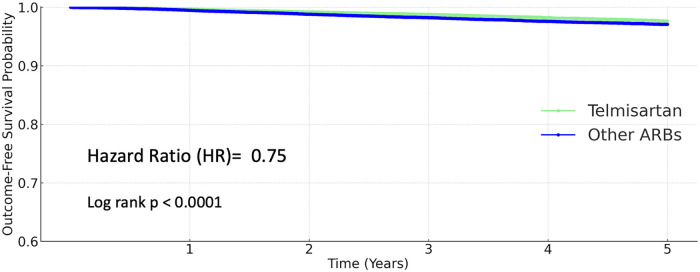
Kaplan–Meier curves for heart failure (telmisartan vs. other ARBs).

During the 5-year follow-up period, heart failure occurred in 801 patients (1.926%) receiving other ARBs and in 540 patients (1.298%) receiving telmisartan. The 5-year cumulative incidence of heart failure was significantly lower in the telmisartan group. Telmisartan was associated with a 25% relative risk reduction compared to other ARBs (HR 0.75, 95% CI 0.672–0.836; *P* < 0.0001; Figure 2).

### Stroke

**Figure 3:**
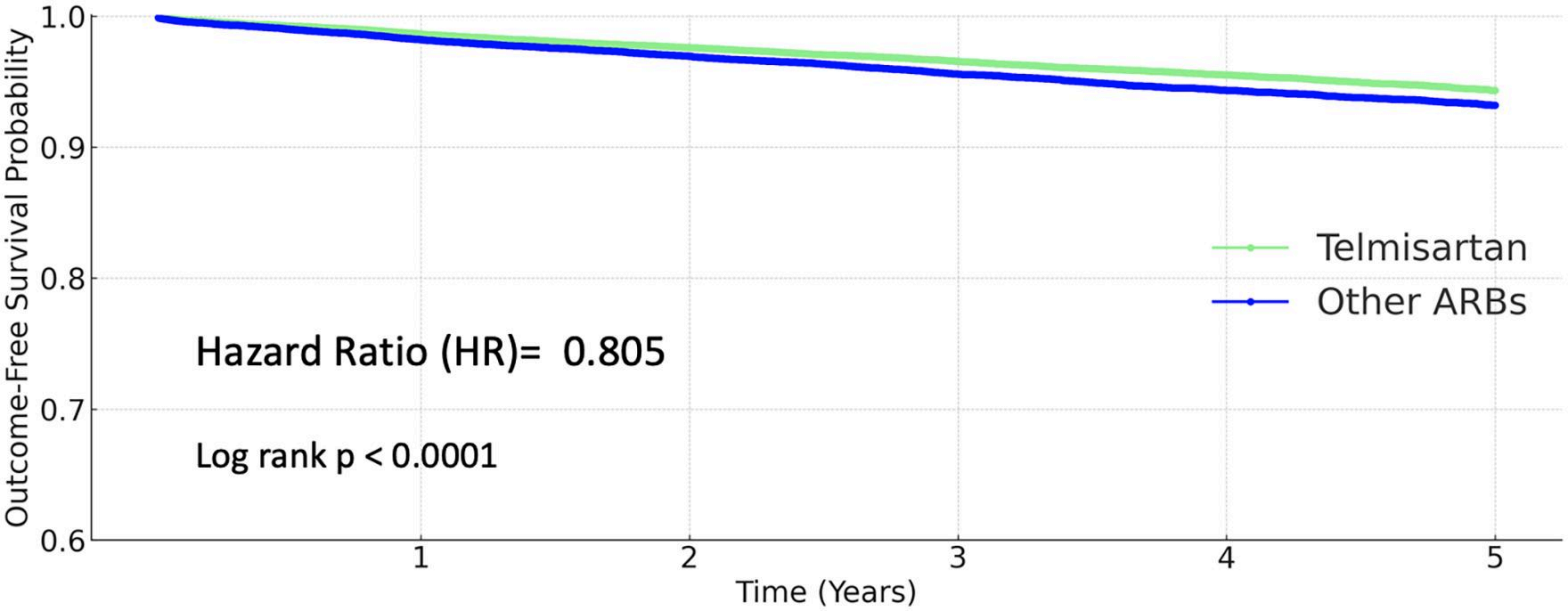
Kaplan–Meier curves for stroke (telmisartan vs. other ARBs).

Among patients receiving other ARBs, 1,868 strokes (4.667%) occurred, compared to 1,383 events (3.439%) in the telmisartan group. Telmisartan use was associated with a significantly lower risk of stroke (HR 0.805, 95% CI 0.751–0.863; *P* < 0.0001; Figure 3).

### Coronary artery disease

**Figure 4:**
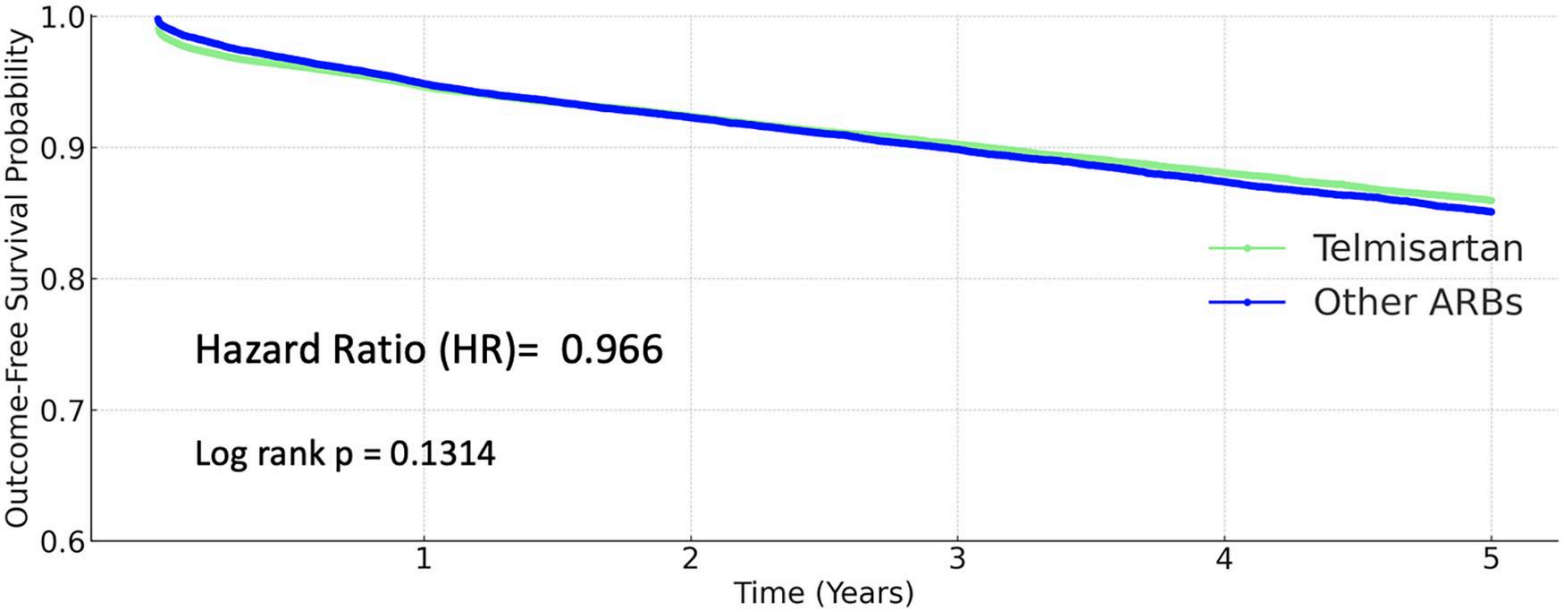
Kaplan–Meier curves for coronary artery disease (telmisartan vs. other ARBs).

A total of 3,924 coronary artery disease events (10.801%) occurred in the other ARB group, and 3,529 events (9.701%) in the telmisartan group. However, the difference was not statistically significant (HR 0.966, 95% CI 0.923–1.011; *P* = 0.1314; Figure 4).

### All-cause mortality

**Figure 5:**
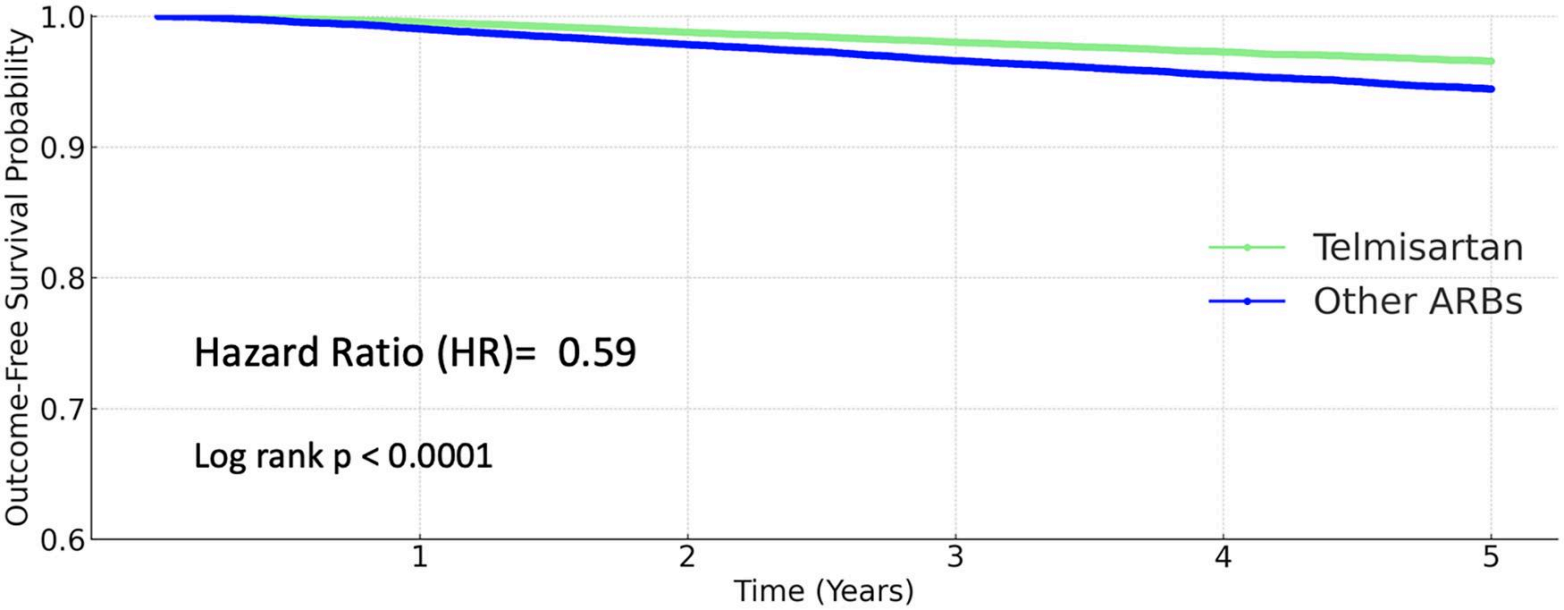
Kaplan–Meier curves for all-cause mortality (telmisartan vs. other ARBs).

Telmisartan use was associated with a markedly reduced risk of all-cause death. The 5-year mortality rate was 3.681% in the other ARBs group and 1.951% in the telmisartan group. The hazard ratio for death with telmisartan was 0.59 (95% CI 0.542–0.642; *P* < 0.0001; Figure 5).

**Figure 6:**
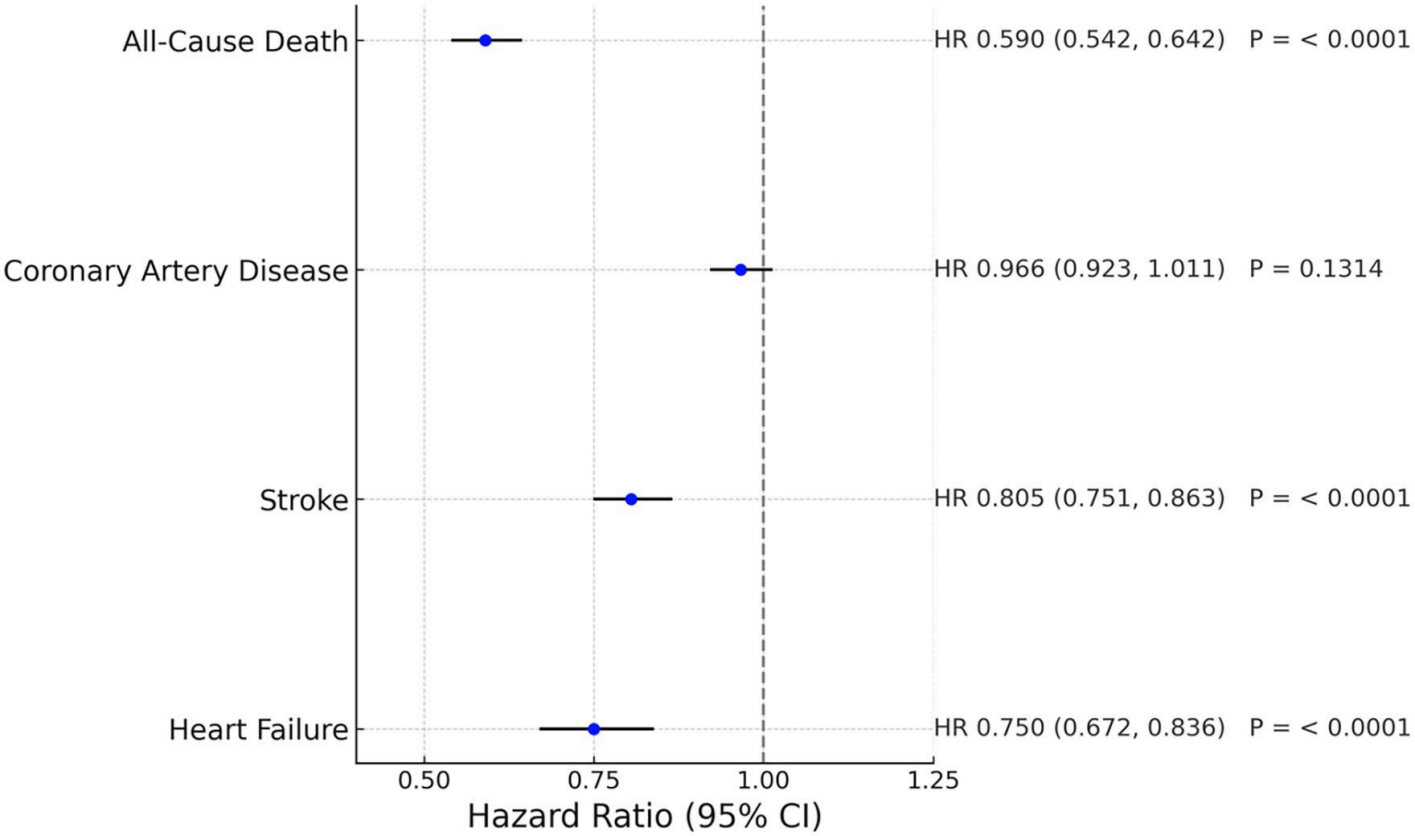
Forest plot of major clinical outcomes comparing telmisartan and other ARBs.

Hazard ratios and 95% confidence interval of all the major outcomes were summarized in a forest plot (Figure 6).

### Subgroup analysis

A sensitive analysis stratified by sex, CKD, hyperlipidemia and diabetes in this cohort is shown in Table 3. In both male and female patients, telmisartan use was associated with reduced risks of stroke, heart failure, and all-cause death (*P* < .05 for both sexes across these outcomes), with no association to coronary artery disease (*P* = ns).

**Table 3:** Subgroup analysis on the risk of major outcomes.

Events	Group	Hazard ratio, 95% CI	*P*-value
Male			
Heart failure			
	Other ARBs	1.0	Ref.
	Telmisartan	0.668 (0.578,0.773)	<0.0001
Stroke			
	Other ARBs	1.0	Ref.
	Telmisartan	0.837 (0.757,0.925)	0.0005
Coronary artery disease			
	Other ARBs	1.0	Ref.
	Telmisartan	0.948 (0.895,1.005)	0.0712
All-cause death			
	Other ARBs	1.0	Ref.
	Telmisartan	0.691 (0.616,0.776)	<0.0001
			
Female			
Heart failure			
	Other ARBs	1.0	Ref.
	Telmisartan	0.711 (0.605,0.835)	<0.0001
Stroke			
	Other ARBs	1.0	Ref.
	Telmisartan	0.847 (0.766,0.937)	0.0012
Coronary artery disease			
	Other ARBs	1.0	Ref.
	Telmisartan	0.94 (0.873,1.012)	0.1024
All-cause death			
	Other ARBs	1.0	Ref.
	Telmisartan	0.638 (0.559,0.729)	<0.0001
Chronic kidney disease			
Heart failure			
	Other ARBs	1.0	Ref.
	Telmisartan	0.736 (0.618,0.877)	0.0006
Stroke			
	Other ARBs	1.0	Ref.
	Telmisartan	0.754 (0.652,0.871)	0.0001
Coronary artery disease			
	Other ARBs	1.0	Ref.
	Telmisartan	0.843 (0.756,0.94)	0.0021
All-cause death			
	Other ARBs	1.0	Ref.
	Telmisartan	0.786 (0.657,0.941)	0.0086
Hyperlipidemia			
Heart failure			
	Other ARBs	1.0	Ref.
	Telmisartan	0.631 (0.555,0.717)	<0.0001
Stroke			
	Other ARBs	1.0	Ref.
	Telmisartan	0.819 (0.756,0.888)	<0.0001
Coronary artery disease			
	Other ARBs	1.0	Ref.
	Telmisartan	0.801 (0.757,0.847)	<0.0001
All-cause death			
	Other ARBs	1.0	Ref.
	Telmisartan	0.71 (0.628,0.804)	<0.0001
Diabetes mellitus			
Heart failure			
	Other ARBs	1.0	Ref.
	Telmisartan	0.768 (0.659,0.895)	0.0007
Stroke			
	Other ARBs	1.0	Ref.
	Telmisartan	0.781 (0.7,0.871)	<0.0001
Coronary artery disease			
	Other ARBs	1.0	Ref.
	Telmisartan	0.906 (0.84,0.978)	0.0114
All-cause death			
	Other ARBs	1.0	Ref.
	Telmisartan	0.704 (0.613,0.808)	<0.0001

**Figure 7:**
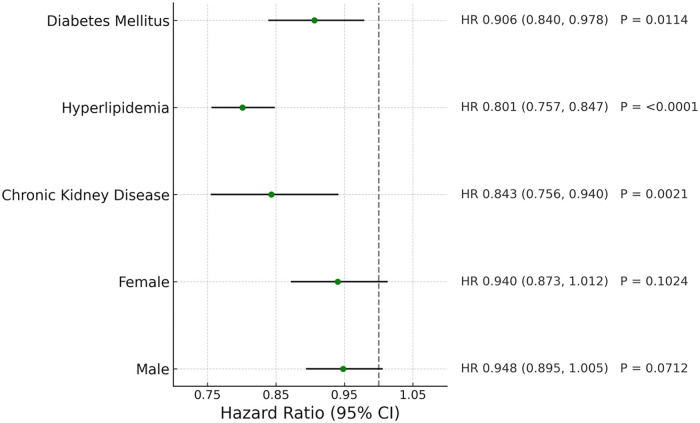
Forest plot of coronary artery disease in different patient groups.

For patients with diabetes, CKD and hyperlipidemia, telmisartan use was associated with reduced risks of stroke, coronary artery disease, heart failure and all-cause mortality as shown in Figure 7 (*P* < .05).

We also conducted a smaller analysis comparing telmisartan with specific drugs. Before propensity score matching, the telmisartan cohort included 44,317 patients, whereas the losartan cohort comprised 1,243,749 patients. After propensity score matching, two balanced cohorts were generated for outcome analysis. In the matched comparison, 1,088 heart failure events occurred in the losartan group and 690 in the telmisartan group (HR 0.699, 95% CI 0.636–0.769). For stroke, 2,289 events occurred among losartan users and 1,684 among telmisartan users (HR 0.794, 95% CI 0.746–0.846). Coronary artery disease was observed in 4,809 patients in the losartan cohort and 4,592 in the telmisartan cohort (HR 1.048, 95% CI 1.007–1.092). For all-cause mortality, 1,906 deaths occurred in the losartan cohort and 887 in the telmisartan cohort (HR 0.512, 95% CI 0.473–0.554) (Table 4).

**Table 4:** Major clinical outcomes comparing telmisartan and losartan.

Events	Total number for analysis	5-year cumulative rate (%)	Hazard ratio, 95% CI	*P*-value
Heart failure				
Losartan	48,688	1,088 (2.235%)	1.0	Ref.
Telmisartan	48,688	690 (1.417%)	0.699 (0.636,0.769)	< 0.0001
Stroke				
Losartan	47,313	2,289 (4.838%)	1.0	Ref.
Telmisartan	47,278	1,684 (3.562%)	0.794 (0.746,0.846)	< 0.0001
Coronary artery disease				
Losartan	44,161	4,809 (10.89%)	1.0	Ref.
Telmisartan	42,901	4,592 (10.704%)	1.048 (1.007,1.092)	0.0223
All-cause death				
Losartan	48,606	1,906 (3.921%)	1.0	Ref.
Telmisartan	48,628	887 (1.824%)	0.512 (0.473,0.554)	< 0.0001

Before propensity score matching, the telmisartan cohort included 44,317 patients, whereas the valsartan cohort comprised 313,188 patients. After propensity score matching, two balanced cohorts were generated for outcome analysis. In the matched comparison, 1,498 heart failure events occurred in the valsartan group and 716 in the telmisartan group (HR 0.480, 95% CI 0.439–0.525). For stroke, 1,991 events occurred in the valsartan cohort and 1,751 in the telmisartan cohort (HR 0.875, 95% CI 0.821–0.933). Coronary artery disease was observed in 4,760 valsartan users and 4,733 telmisartan users (HR 0.980, 95% CI 0.941–1.020). For all-cause mortality, 1,516 deaths occurred in the valsartan cohort and 900 in the telmisartan cohort (HR 0.600, 95% CI 0.552–0.651) (Table 5).

**Table 5:** Major clinical outcomes comparing telmisartan and valsartan.

Events	Total number for analysis	5-year cumulative rate (%)	Hazard ratio, 95% CI	*P*-value
Heart failure				
Valsartan	50,309	1,498 (2.978%)	1.0	Ref.
Telmisartan	50,309	716 (1.423%)	0.48 (0.439,0.525)	< 0.0001
Stroke				
Valsartan	48,134	1,991 (4.136%)	1.0	Ref.
Telmisartan	48,841	1,751 (3.585%)	0.875 (0.821,0.933)	< 0.0001
Coronary artery disease				
Valsartan	43,334	4,760 (10.984%)	1.0	Ref.
Telmisartan	44,393	4,733 (10.662%)	0.98 (0.941,1.02)	0.3259
All-cause death				
Valsartan	50,256	1,516 (3.017%)	1.0	Ref.
Telmisartan	50,249	900 (1.791%)	0.6 (0.552,0.651)	< 0.0001

## Discussion

Telmisartan is distinguished among angiotensin II receptor blockers (ARBs) by its high lipophilicity and long elimination half-life, allowing for sustained 24-hour blood pressure control. This pharmacokinetic advantage is particularly relevant for mitigating early morning blood pressure surges, which are known to increase the risk of cerebrovascular events such as stroke (23–25). In addition, telmisartan acts as a partial agonist of peroxisome proliferator-activated receptor gamma (PPAR-γ), a nuclear receptor involved in glucose and lipid metabolism. This pleiotropic activity has been linked to anti-inflammatory effects, endothelial stabilization, and improved insulin sensitivity, which may contribute to both neuroprotective and cardioprotective benefits (7, 26–32).

In our study, telmisartan use was associated with significantly lower risks of stroke, heart failure, and all-cause mortality compared to other ARBs. These associations were consistent across key subgroups, including patients with diabetes, chronic kidney disease, and hyperlipidemia—populations in which telmisartan's pleiotropic mechanisms may offer additional clinical value. The large, real-world nature of our cohort, comprising over 1.9 million patients and a well-matched analytic sample of 41,598 individuals in each treatment group, enhances the generalizability of our findings. Moreover, the consistency of the observed treatment effects across diverse clinical strata strengthens the internal validity of the results and suggests that telmisartan may confer benefits beyond blood pressure reduction alone.

In the ARB-specific analyses comparing telmisartan with losartan and valsartan, the overall pattern of hazard ratios for heart failure, stroke, and all-cause mortality was directionally similar across both comparisons, with consistently lower estimates observed in the telmisartan cohorts. The primary distinction between the two analyses emerged in the coronary artery disease outcome. In the telmisartan–losartan comparison, the hazard ratio for coronary artery disease was slightly above unity (favoring losartan), whereas in the telmisartan–valsartan comparison the estimate was close to 1.0 with no statistically significant difference. This divergence suggests that the coronary artery disease findings may vary depending on the specific ARB comparator, potentially reflecting differences in prescribing patterns, population characteristics, or residual confounding that persists in real-world data despite matching. Nevertheless, when viewed together, the two comparisons provide a broader context for interpreting how telmisartan performs relative to individual ARBs in routine clinical settings.

Our findings also align with several landmark clinical trials of telmisartan. The ONTARGET trial demonstrated that telmisartan was non-inferior to ramipril in reducing major cardiovascular events in patients with vascular disease or diabetes with end-organ damage and was associated with fewer adverse effects and better treatment adherence (12). Similarly, the TRANSCEND trial, which focused on ACE inhibitor–intolerant patients, showed that telmisartan was well-tolerated and modestly reduced the risk of the composite outcome of cardiovascular death, myocardial infarction, or stroke (13). The PRoFESS trial, which evaluated telmisartan for secondary stroke prevention, did not find a significant reduction in recurrent stroke risk (33). However, the PRoFESS population included patients with recent ischemic stroke, whereas our cohort excluded individuals with a history of stroke or cardiovascular disease. This key difference in baseline risk may help explain the discrepancy in outcomes and suggests that telmisartan may be more effective in primary prevention settings or among patients without established cerebrovascular disease.

Other trials have further explored telmisartan's renal and cardiovascular effects. In the AMADEO trial, telmisartan demonstrated superior efficacy to losartan in reducing proteinuria among patients with diabetic nephropathy (34, 35). The DETAIL study found that telmisartan and enalapril offered comparable long-term renal protection in type 2 diabetic patients with early nephropathy (36, 37). Despite heterogeneity in trial populations and endpoints, a consistent finding across all major studies—including our own—is telmisartan's favorable safety and tolerability profile. Due to the smaller patient numbers in the telmisartan cohorts and possible differences between cohorts, the generalizability of our findings may be somewhat limited. Nevertheless, these results provide real-world evidence and the potential wider use of telmisartan and might guide future well-controlled clinical trials.

Nonetheless, several limitations inherent to our retrospective, observational design should be acknowledged. First, although propensity score matching was employed to minimize baseline differences, residual confounding from unmeasured variables, such as frailty, socioeconomic status, and baseline blood pressure control, was not accounted for. Second, our use of data from the TriNetX platform—while extensive—relies on the accuracy and completion of electronic health records, which may vary across participating institutions. Third, detailed information on medication dosage, duration of therapy, and adherence was not available in the structured dataset, precluding dose-response analyses or evaluations of cumulative exposure. Fourth, the relatively small population of telmisartan-using patients added more confounding to the comparison between telmisartan and other drugs in this genre.

## Conclusion

In this large-scale, real-world cohort study leveraging the TriNetX Global Collaborative Network, we evaluated cardiovascular outcomes in more than 1.9 million patients with hypertension and identified 41,598 well-matched individuals in each treatment group. Telmisartan use was consistently linked to reduced risks of stroke, heart failure, and all-cause mortality compared with other ARBs. These findings, observed across key clinical subgroups, suggest that telmisartan may offer incremental cardiovascular and cerebrovascular benefits beyond blood pressure control. While further prospective studies are needed to confirm causality, our results support consideration of telmisartan as a potentially advantageous therapeutic option for hypertensive patients at elevated cardiovascular risk.

## Data Availability

The data that support the findings of this study are available from the TriNetX Global Health Research Network. Restrictions apply to the availability of these data, which were used under license for the current study and are not publicly available. Data may be available from the authors upon reasonable request.
